# Observations of Wart Clearance Following COVID-19 Vaccination: Coincidence or Missed Immunologic Signals?

**DOI:** 10.3390/vaccines13111081

**Published:** 2025-10-22

**Authors:** Qiwei Wilton Sun, Caroline A. Nelson, Howard P. Forman

**Affiliations:** 1 Yale School of Medicine, 333 Cedar Street, New Haven, CT 06510, USA; 2 Department of Dermatology, Yale School of Medicine, New Haven, CT 06510, USA; 3 Department of Radiology and Biological Imaging, Yale School of Medicine, New Haven, CT 06510, USA; howard.forman@yale.edu; 4 Yale School of Management, New Haven, CT 06511, USA; 5 Yale School of Public Health, New Haven, CT 06510, USA

**Keywords:** COVID-19, vaccines, HPV, warts, dermatology

## Abstract

**Background/Objectives:** The COVID-19 vaccines have been extensively studied for their potential adverse side effects. However, reports of unexpected but potentially beneficial immune responses have received comparatively less attention. **Methods:** In this case series, a PubMed search was conducted using the terms “warts”, “verruca”, “HPV”, “COVID-19”, “SARS-CoV-2”, “immunization”, and “vaccination.” All reported cases of wart clearance temporally linked to COVID-19 vaccination were identified and summarized, including patient demographics, vaccine type, number of doses, timing of clearance, and follow-up duration. **Results:** Five cases were identified. Patients varied in age, sex, comorbidity, and immunologic status. Warts were long-standing and treatment-resistant in all cases. Clearance occurred within approximately 2–4 weeks following the second or third vaccine dose (either mRNA-based [BNT162b2, mRNA-1273] or adenoviral vector [ChAdOx1-S]) and was sustained for 2–8 months of follow-up with no recurrences reported. **Conclusions:** While causality cannot be determined, the convergence of reports across diverse patients, consistent timing of clearance, and plausible immunologic pathways suggest that COVID-19 vaccination may, in rare instances, trigger beneficial immune activation against HPV-infected keratinocytes. Recognition of such unexpected outcomes underscores the need for broader vaccine safety and efficacy surveillance that includes both adverse and beneficial immune effects.

## 1. Introduction

The unprecedented speed and scale of deployment of the COVID-19 vaccines have prompted considerable interest in understanding their associated side effects, with numerous cutaneous side effects reported. The most common categories include early local injection-site reactions [1], urticaria and angioedema [2], morbilliform and other exanthematous eruptions [3], and reactivation of herpes zoster [4]. Vaccines are traditionally evaluated by their efficacy in preventing infections, reducing clinical severity, and ensuring a robust safety profile; as such, the vigilance for adverse effects is justified. However, the focus on risks may conversely overlook the possibility that vaccines may also elicit unexpected yet beneficial immunologic responses that fall outside of traditional endpoints.

This article was motivated by difficult-to-explain observations of clearance of long-standing, treatment-resistant warts following COVID-19 vaccination. Warts are benign proliferations of human papillomavirus (HPV)-infected keratinocytes that can be difficult to treat due to partial response to treatment and high recurrence rates. Their resistance is hypothesized to stem from HPV-mediated evasion from and downregulation of the host immune system, creating an immune-privileged environment [5]. Localized immune activation therapies, including intralesional Candida antigen, intralesional Measles-Mumps-Rubella (MMR), and topical imiquimod, enhance immune recognition and clearance of HPV-infected keratinocytes. While only speculative, reports of wart clearance temporally associated with COVID-19 vaccination raise the possibility of a vaccine-induced immune effect. Here, we present a cluster of such cases, and we discuss the existing precedence for such observations, potential mechanisms, and the importance of broadening the scope of vaccine effects considered worthy of further investigation. This report is intended to be descriptive and hypothesis-generating, contextualizing independent clinical observations rather than asserting causation.

## 2. Case Presentations

We conducted a literature search in PubMed to identify all reported cases of wart clearance following COVID-19 vaccination to date using combinations of the terms “warts”, “verruca”, “HPV”, “COVID-19”, “SARS-CoV-2”, “coronavirus”, “immunization”, and “vaccination”. We identified five total cases, herein presenting each case in a standardized format, highlighting patient demographics, wart history, vaccine type and number of doses, timing of clearance, notable features of the case if applicable, and follow-up. These cases are summarized in Table 1.

### 2.1. Case 1

A 28-year-old woman with hypothyroidism first developed multiple verrucous papules on the palmar aspect of the distal right thumb and base of the right index finger 2 years ago that regrew despite mechanical removal and over-the-counter freezing spray [6]. She received two doses of the ChAdOx1-S (AstraZeneca^®^) vaccine in March 2021–April 2021. Notably, the patient experienced increased hair loss after each dose and began taking biotin 2 weeks after the second dose. One week after taking biotin, and 3 weeks after the second dose, she experienced crust formation and pain with her lesions, and complete clearance of lesions four weeks after the second dose. The last date of recorded follow-up was 4 months post-vaccination, at which point her lesions remained cleared.

### 2.2. Case 2

A 12-year-old healthy female first developed hyperkeratotic papules confined in the periungual aspects of the fingers 4 years ago [7]. The lesions did not clear with treatment with topical formic acid and cryotherapy. She received two doses of the mRNA BNT162b2 vaccine (Pfizer-BioNTech^®^) in September 2021–October 2021, and experienced complete clearance of lesions three weeks after the second dose with no therapeutic intervention. The last date of recorded follow-up was June 2022, 8 months post-vaccination, and lesions remained cleared.

### 2.3. Case 3

A 77-year-old woman with unspecified immunosuppression, thrombotic thrombocytopenic purpura in remission, macular degeneration, hypothyroidism, hypertension, and dyslipidemia presented with dome-shaped, hyperkeratotic papules localized to the distal interphalangeal joint for 8 years [7]. The patient had not been previously treated for the warts. She received three doses of the mRNA BNT162b2 vaccine in March, April, and October 2021, and experienced significant regression of lesions 4 weeks after the third booster dose. At the last date of follow-up in June 2022, 8 months post-vaccination, no lesion recurrence occurred.

### 2.4. Case 4

A 27-year-old male with no reported medical history had multiple verrucous coalescing papules around the mandible and bilateral temples for 1 year that recurred despite multiple sessions of radiofrequency ablation [8]. He received two doses of the ChAdOx1-S vaccine and, 10 days after the second dose, self-reported flattening of lesions, with regression of almost all lesions over the next week. The timing of the last date of recorded follow-up was approximately 2 months post-vaccination, with no recurrences reported.

### 2.5. Case 5

A 63-year-old male with HIV on abacavir/lamivudine and darunavir/cobicistat and treated Hepatitis C previously on ledipasvir/sofosbuvir presented with extensive hyperkeratotic, dome-shaped, verrucous papules on the bilateral metacarpophalangeal joint of the thumbs, and his feet, for 10 years [9]. Notably, these lesions were recalcitrant to cryotherapy, topical 5-fluorouracil, topical imiquimod, intralesional bleomycin, topical cidofivir, and acitretin. He is specifically documented to have no previous history of SARS-CoV-2 infection. He received two doses of the mRNA-1273 (Moderna^®^) vaccine in March–April 2021, and 2 weeks after the second dose, exhibited complete and sustained clearance of all lesions, with the last date of recorded follow-up in December 2021, 8 months post-vaccination.

## 3. Discussion

Across all five published cases of wart clearance following COVID-19 vaccination, the lesions were long-standing and resistant to multiple treatment modalities. Yet, clearance occurred in a similar timeframe of roughly 2–4 weeks following the second or third dose of COVID-19 vaccination, with both mRNA-based and adenoviral vector viruses implicated, and no lesions recurring in the follow-up period. The patients involved varied widely in age, gender, comorbidities, immunologic status, and location and morphology of wart lesions. Importantly, the follow-up duration was inconsistent across cases, and SARS-CoV-2 infection history was not uniformly reported. While causation or quantitative association cannot be asserted solely from these case reports, the synthesis of these existing reports provides an important descriptive foundation for future controlled studies exploring potential immunologic links.

A precedent exists for the observation of cutaneous lesion clearance following infection or vaccination. At least two cases of wart clearance following SARS-CoV-2 infection have been documented [10,11], with both patients exhibiting treatment-resistant warts and notably being organ transplant recipients. In one case, the warts regrew 2 months after infection [10]. In the other case, the individual had received three doses of the COVID-19 vaccine 5 months prior to infection, where no wart clearance was reported [11]. The observation of wart clearance in both natural infection and vaccination suggests the possibility of an underlying immunologic mechanism as both contexts stimulate systemic immune activation [12,13]. Yet, the disparate clinical courses of these cases suggests that any potential underlying immunologic mechanism mediating wart clearance from SARS-CoV-2 infection or COVID-19 vaccine is heterogeneous. Other cutaneous lesions reported to clear incidentally following COVID-19 infection or vaccination include molluscum contagiosum and lichen planus pemphigoides [14,15]. Recent prospective studies evaluating intramuscular HPV vaccine for treatment of existing warts were moderately efficacious, with approximately 45–63% clearance rates [16,17,18]. Though not currently approved for treatment, the preliminary evidence points to a recurring observation where diverse immunogenic stimuli incidentally precede the clearance of chronic cutaneous viral infections.

Varying hypotheses have been proposed to explain the observations of wart clearance following COVID-19 vaccination. Plasmacytoid dendritic cells (pDCs) have been proposed as a key mediator [19]. pDCs are activated by viral antigens, resulting in production of type 1 interferons (IFN-Is) along with broader cytokine responses (IL-1, TNF-a, IL-6, IL-12) [20,21]. Additionally, pDCs and IFN-Is have been shown to play a key role in wart clearance [19,22,23]. While SARS-CoV-2 infection is a key activator of pDCs [24,25], mRNA and adenoviral vector COVID-19 vaccination do not stimulate pDCs to the extent as natural infection [26,27], suggesting this mechanism may be less plausible in the post-vaccination setting. Alternative hypotheses, including a nonspecific “immune boost” similar to the immunotherapeutic effect observed with intralesional MMR or Bacillus Calmette–Guérin (BCG) vaccines, which upregulate Th1 cytokines (↑ IL-1, ↑ IFN-γ, ↓ IL-10) [28,29], have also been proposed. In addition, HPV vaccine-induced systemic immune activation may restore local cutaneous immune surveillance [30,31], leading to recognition and clearance of HPV-infected keratinocytes. While this mechanism has not yet been demonstrated for COVID-19 vaccines, similar pathways may underlie wart clearance following vaccination. Alternatively, wart regression may reflect immune reconstitution phenomena independent of vaccination, such as recovery from transient immunosuppression or broader shifts in immune surveillance capacity. However, given the rarity of reported cases, these mechanisms are speculative and require further study.

Importantly, spontaneous wart clearance is common [32], and the possibility of coincidental timing rather than a true vaccine-mediated effect cannot be ruled out. Paradoxically, at least one purported case of wart emergence following COVID-19 vaccination has also been reported [33]. It also remains unclear why wart clearance is not temporally related to other vaccines, highlighting the importance of caution in interpreting these reports. These findings cannot substitute for a systematic analysis; rather, they serve to generate hypotheses and guide the design of future studies. However, these cases matter not in what they confirm, but in what they prompt us to consider. They reveal potential blind spots in our understanding of vaccine–immune system interactions that reflect broader tendencies to overlook potentially beneficial post-vaccine effects, despite our rightful vigilance for adverse ones. Historical examples, such as the use of BCG vaccine as immunotherapy for bladder cancer, also began with unusual clinical observations later confirmed through study [34]. Not all meaningful immune responses will conform to predefined endpoints, nor will all observations lead to therapeutic applications. Future work should not only confirm or refute causality in these cases but also expand the framework for evaluating vaccines to include a wider range of potential immune-mediated outcomes that would be lost opportunities if ignored.

## 4. Conclusions

In summary, we present five cases of clearance of long-standing, treatment-resistant warts temporally associated with COVID-19 vaccination, occurring in patients of diverse ages, comorbidities, and immunologic statuses. While we re-emphasize that causality cannot be asserted from these difficult-to-explain reports, the consistent timing of clearance followed by periods of sustained clearance across the cases, established precedence in the literature for such observations, and plausible immunologic link warrants further exploration of these observations. The present cases further highlight the importance of considering the full spectrum of vaccine side effects, including unexpected but potentially beneficial responses, such that potentially meaningful observations are not overlooked.

## Figures and Tables

**Table 1:** Reported cases of warts clearance following COVID-19 vaccination.

Age, Gender	Immuno-Compromised?	Location of Lesions ^d^	Duration of Lesions (Years)	Previous Therapies	Interim Medical History Changes	Vaccine Received	Dose Triggering Clearance	Time to Onset of Clearance ^a^	Duration of Sustained Clearance (Months) ^b^
28, Female [6]	No	Thumb, fingers	2	Mechanical self-removal, over-the-counter freezing spray	Began taking biotin 2 weeks after second dose	ChAdOx1-S (AstraZeneca^®^, Cambridge, UK)	2	3 weeks	4
12, Female [7]	No	Periungual, hands	4	Formic acid, cryotherapy	N/A	mRNA BNT162b2 (Pfizer-BioNTech^®^, New York, NY, USA)	2	3 weeks	8
77, Female [7]	Yes (unspecified)	Fingers, hands	8	Untreated	N/A	mRNA BNT162b2 (Pfizer-BioNTech^®^)	3 ^c^	4 weeks	8
27, Male [8]	No	Mandible, temple, neck	1	Radiofrequency ablation	N/A	ChAdOx1-S (AstraZeneca^®^)	2	10 days	3
63, Male [9]	Yes (HIV)	Hands, periungual, feet	10	Cryotherapy, topical 5-fluorouracil, topical imiquimod, intralesional bleomycin, topical cidofovir, acitretin	N/A	mRNA-1273 (Moderna^®^, Cambridge, MA, USA)	2	2 weeks	8

Abbreviations: HIV, human immunodeficiency virus. ^a^ Denotes time from administration of the last dose of vaccine to onset of clearance of lesions. ^b^ Denotes time from administration of last dose of vaccine to last reported follow-up date. ^c^ Booster dose triggering wart clearance. ^d^ For images of lesions before and after vaccination, please refer to each of the cited original case reports: https://doi.org/10.1111/jdv.17771; https://doi.org/10.1111/jdv.18577; https://doi.org/10.1016/j.jdin.2022.02.007; https://doi.org/10.1016/j.jdcr.2022.11.007.

## Data Availability

The original data presented in the study are openly available in PubMed at: [https://doi.org/10.1111/jdv.17771; https://doi.org/10.1111/jdv.18577; https://doi.org/10.1016/j.jdin.2022.02.007; https://doi.org/10.1016/j.jdcr.2022.11.007].
